# Structural characterization of poly-Si Films crystallized by Ni Metal Induced Lateral Crystallization

**DOI:** 10.1038/s41598-019-39503-9

**Published:** 2019-02-26

**Authors:** N. Vouroutzis, J. Stoemenos, N. Frangis, G. Z. Radnóczi, D. Knez, F. Hofer, B. Pécz

**Affiliations:** 1 Department of Physics, Aristotle University of Thessaloniki, GR-54124 Thessaloniki, Greece; 2 Institute for Technical Physics and Materials Sci., Centre for Energy Research, Hungarian Academy of Sciences, MTA EK MFA, 1121 Budapest, Konkoly-Thege M. u. 29-33, Hungary; 3 Institute for Electron Microscopy and Nanoanalysis & Graz Centre for Electron Microscopy, TU Graz, Steyrergasse 17, 8010 Graz, Austria

## Abstract

The growth of the poly-Si films was studied by Transmission Electron Microscopy (TEM) after Ni Metal Induced Lateral Crystallization (Ni-MILC) of amorphous Si films at 413 °C. Significant differences in the morphology and the mode of growth of the films were observed, in comparison to films grown at temperatures above 500 °C. It was shown that at 413 °C the Solid Phase Crystallization (SPC), which acts in parallel with the Ni-MILC process at temperatures above 500 °C is suppressed. The suppression of SPC results in substantial change in the mode of growth. The poly-Si film grown at 413 °C consists of whiskers, which can be classified into two categories. Those growing fast along the <111> direction, which were already observed in conventional Ni-MILC above 500 °C and whiskers grown along random crystallographic orientations having significantly slower growth rates. Because of the large difference in growth rates of the whiskers, significant orientation filtering due to growth-velocity competition is observed. The uniform poly-Si films consist of a mixture of fast <111> type whiskers and slow ones, grown in other orientations, resulting in a tweed-like structure.

## Introduction

Polycrystalline silicon (poly-Si) films are used in a wide range of applications, like in large-area electronics, including thin-film transistors (TFTs), solar cells and sensors. Solid phase crystallization (SPC) is the most common method to crystallize amorphous silicon (*a*-Si) films despite the inconveniently high operation temperatures above 600 °C. It is known that metals, like aluminum, nickel, gold, silver will lower the SPC temperature of *a*-Si^1^. Therefore, Metal Induced Crystallization (MIC) is a process in which a metal promotes crystallization of the amorphous semiconductor at low temperature. Nickel is often preferred over other metals because electric parameters of the resulting poly-Si are less disturbed by nickel than by other metals^2^. The reaction between the Ni and *a*-Si occurs at an interlayer by diffusion and it lowers the crystallization temperature. This enhancement is due to the change in the covalent bonds at the interface caused by their interaction with the free electrons from the metallic phase^3^.

In the Ni/*a*-Si system, the thermodynamically favored phase NiSi_2_ is formed at 280 °C^4^ or lower^5^. Hayzelden and Batstone^6^ have demonstrated the formation of NiSi_2_ precipitates by ion implantation of Ni into *a*-Si followed by heat treatment.

**Figure 1:**
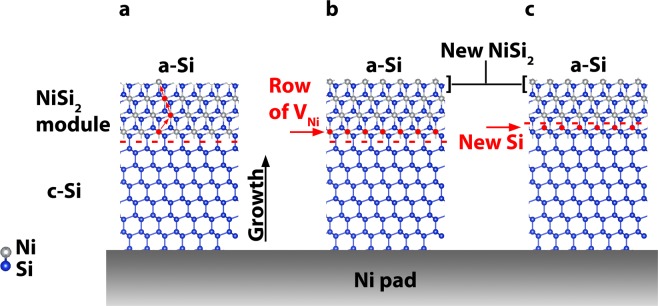
Model of the MILC process. (**a**) Nickel atoms from the crystalline NiSi_2_ diffuse to the a-Si leaving Ni vacancies behind. (**b**) New NiSi_2_ is formed inside the a-Si in front of the of the Nickel-disilicide grain. (**c**) After Si-bonds rearrange at the Ni-vacancies crystalline Si is formed at the c-Si/NiSi_2_ interface.

Nickel-disilicide (NiSi_2_) is cubic having CaF_2_ structure and lattice mismatch with crystalline Si of only 0.4%, can be formed at temperatures as low as 250 °C. The Si crystallization starts preferentially at the {111} facets of the NiSi_2_ grains. Nickel atoms from the Ni-disilicide grain move into the a-Si forming there NiSi_2_, in parallel, the backside of the NiSi_2_ module is accumulated in Ni vacancies at the c-Si/NiSi_2_ interface resulting in diamond type rearrangement of the Si bonds, forming a new Si epitaxial crystalline layer at the backside of the NiSi_2_ module, as shown in the atomic scale schematic representation in Fig. 1a–c. By repeating this process, long Si whiskers are formed. Ni migration into a-Si is driven by the difference of the free energy between metastable a-Si and the stable c-Si^6^. It is worth noticing that the number of Si atoms in the NiSi_2_ and Si unit cells is the same, and the only species which is diffusing in this process is Ni. The Ni–MILC is based on the formation of nickel-disilicide pads and the one-dimensional migration of their fragments^7,8^.

In the standard MILC process above 500 °C the crystallization occurs via the growth of whiskers, however, with the contribution of conventional SPC process as well. In the present work, we have focused to the structural characteristics of the poly-Si films grown at 413 °C where the SPC process is suppressed. To follow the morphology as well as the processes at atomic level, conventional TEM, High Resolution TEM (HRTEM) and High Resolution Scanning-TEM (HRSTEM) are used.

## Results and Discussion

### Poly-Si films formed by Ni-MILC in the range of temperatures 500–600 °C

**Figure 2:**
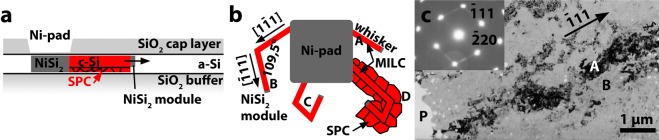
The evolution of the crystalline phase during MILC process. (**a**) Cross section of a thin, crystallizing Si film near the Ni pad. Small NiSi_2_ modules start moving into the a-Si leaving a crystalline Si trail behind. Further crystallization occurs at the boundaries of the crystalline phase by SPC. (**b**) Schematic representation in plain view of the <111> Si whiskers which are formed around a NiSi_2_ pad. The whiskers can change direction to another equivalent <111>, these are denoted by the letters B and C. The combined MILC and SPC process is shown in the whisker D. (**c**) low magnification PVTEM micrograph shows the formation of large grains around the NiSi_2_ pads exhibiting [110] preferred orientation as shown by the Selected Area Diffraction in the inset, which was taken from the grain A. The grain exhibits obscure GBs and black white mottle-like contrast, characteristic in mosaic structures.

For the standard Ni-MILC process above 500 °C *(in contrast to our experiments carried out at lower temperatures)*, at first thin (about 50 nm thick) Ni pads are formed by lithography on the *a*-Si film^5^. After that, the *a*-Si films are annealed in the range of temperatures 500 to 600 °C for the formation of poly-Si^9^. Si whiskers are formed preferentially on the {111} NiSi_2_ facets^6^ with a typical growth direction of <111>. Ideally the <111> direction of a NiSi_2_ grain defining the growth direction lays in the film plane and {111} planes perpendicular to it as shown in Fig. 2a. Si-whiskers of different, inclined growth directions very soon reach the bottom or the top of the film and terminate^10,11^. Whiskers often form kinks and change growth direction to another equivalent <111> direction, which may also be parallel to the film plane, as shown in Fig. 2b. In this way, the whiskers form a net gradually covering the former *a*-Si film area by growing along all the equivalent <111> directions. The remaining amorphous silicon is crystallized by conventional SPC with the whiskers taking the role of seeds (Fig. 2b). Finally, large V-shape grains with obscure grain boundaries exhibiting strong preferred (110) orientation are formed around the pad due to these two mechanisms^5^.

The grains which are formed by the Ni-MILC process at 600 °C exhibit grain boundaries with irregular shape as shown in the grain denoted by the letter A in Fig. 2c. In general, the grains show high contrast variations in bright field imaging characteristic to mosaic structures; this also explains the obscure GBs^5^. The misoriented sub-grains have a mean size of 60 nm. Earlier studies revealed misorientations in overlapping subgrains up to 8° resulting in Moiré patterns^5,12^.

It is known^9^ that contribution of SPC to Ni-MILC above 500 °C is substantial, therefore, conventional Ni-MILC must be considered above this temperature. It also alters the growth mode as revealed by *in-situ* heating experiments^11^. At first glance the contribution of SPC should be insignificant because the incubation time for spontaneous nucleation at 600 °C is more than 10 hours^13^, whereas there is no such delay in the case of Ni-MILC. In addition, Ni-MILC produces 50 times faster crystallization rate at 600 °C (about 18 µm/hour), compared to SPC^14^. However, the Si-whiskers act as nucleation sites ruling out the long SPC incubation period making its contribution in Ni-MILC significant, as schematically shown in the inset in Fig. 2b. The SPC contribution in MILC was clearly observed in annealing series, details are given in Supplementary information. The sub-grains can be eliminated by subsequent Excimer Laser Annealing (ELA)^10^. It is worth noticing that the standard SPC of *a*-Si at 600 °C, is mediated by twin formation^15^ as twins promote faster growth of Si grains. The c-Si obtained by SPC exhibits (111) type micro-twins with a relatively high density of the order of 5 × 10^12^ cm^−2^^16^.

### Poly-Si films formed by Ni-MILC at 413 °C

**Figure 3:**
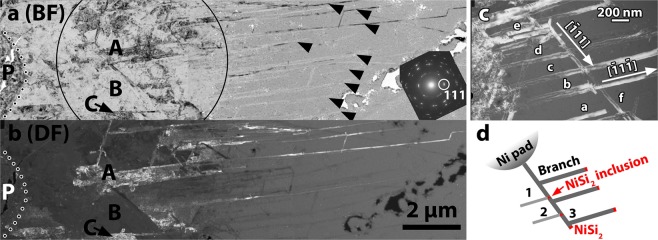
PVTEM micrographs from a poly-Si film after Ni-MILC at 413 °C for 32 Ds. (**a**) BF overview. The Pad is denoted by the letter P. The inset is the SAD pattern taken from the area denoted by the circle, which includes the continuous crystalline film and part of the long whiskers. (**b**) Tilted DF of the same area taken from the 111 diffraction spot shown in the inset in figure (**a**). (**c**) Formation of parallel branch whiskers of <111> type. (**d**) Schematic representation either by double generations from the NiSi_2_ module or the by tetrahedral NiSi_2_ inclusions.

Only pure Ni-MILC occurs at this low temperature, the SPC process is completely inhibited. Consequently, the morphology and the mode of growth of the films also changed. The films consist of a mixture of fast growing <111> type whiskers and slow ones having random crystallographic orientations other than <111>. The overview of the layer annealed at this temperature for 32 Ds is shown in the Bright Field (BF) and Dark Field (DF) micrographs in Fig. 3a,b, respectively. The micrographs contain the NiSi_2_ pad denoted by the letter P, the continuous poly-Si film including large Si grains denoted by the letters A, B, and C, as well as the long parallel whiskers shown by arrows, which extend deep inside the a-Si. The 111 reflection highlighted in the inset of Fig. 3a was selected to take the dark field image shown in Fig. 3b. The diffraction pattern was taken with a large SAD aperture including the grains A, B and C as shown by black circle in Fig. 3a. The continuous film consists of a net of long and short whiskers resulting in a tweed-like structure. The large grains denoted by the letter A and C, as well as the long parallel whisker are grown along the [111] direction, as shown in Fig. 3a,b, revealing that have the same origin. Most of the long whiskers have a width of 60 nm which is the mean size of the NiSi_2_ grains in the pad.

In some cases, whiskers intersect with other ones having a different growth direction but with a common lattice, revealing their common origin. This is the case of the parallel [111] type whiskers in section (110), which are denoted by the letters a, b, c, d, they intersect the [111] whisker f, without interruption as shown in Fig. 3c. The common origin of the parallel whisker a, b, c, d, e, can be explained by considering that all of them are branches of a parental whisker. The branch whiskers were created along the equivalent <111> direction, by the leading NiSi_2_ module during its movement, as schematically shown in Fig. 3d. This mechanism explains the origin of the parallel whiskers shown by arrows in Fig. 3a,b. The generation of parallel parental whiskers is the characteristic growth of the low temperature Ni-MILC.

The formation of the long <111> whiskers is the result of the growth-velocity competition. This effect is also observed in poly-Si films grown by SPC^16^, due to the difference in the crystallization rate versus crystallographic orientation in Si^17^.

**Figure 4:**
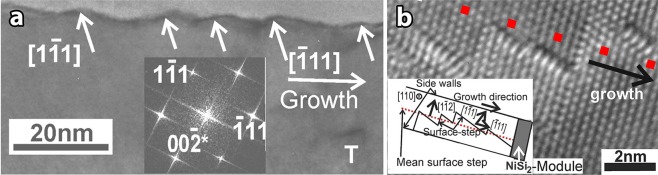
PVTEM micrographs from whiskers after Ni-MILC at 413 °C for 32 Ds. (**a**) Saw-tooth faceting of the sidewall of a [111] type whisker in (220) section. One family of the facets is perpendicular to the [111] direction, the other set is almost perpendicular to the [001], as it is confirmed by the corresponding FFT in the inset. A V-shape defect having branches along the (111) and the (111) planes is denoted by the letter T. (**b**) A surface step on the (220) surface plane was formed by the advancing NiSi_2_ module, perpendicular to the [111] growth direction of the whisker, denoted by the red dotted line. In the reality the step consist of nano-steps along the [111] and [111] directions. The overall representation is shown schematically in the inset.

The whiskers retain their width constant due to the absence of SPC at the sidewalls, resembling Vapor-Liquid-Solid (VLS) growth^18^. A second similarity of the low temperature Ni-MILC in respect to VLS is the saw-tooth faceting of the sidewalls of the whiskers as shown in Fig. 4a. The corresponding Fast Fourier Transform (FFT) in the inset of Fig. 4a reveals that one side of the tooth is the (111) facet, the other side, not so sharp, is faceting close to [001] direction. Faceting occurs when the sidewalls parallel to the direction of crystallization are not stable, i.e. their shape is different from the equilibrium one^19^. In Si the equilibrium crystal shape is the octahedral one, bounded by eight {111} facets. Therefore, for a whisker crystallized along the [111] direction there are no stable facets parallel to this direction. This instability is also confirmed by the surface steps on the (220) surface plane of a whisker grown along the [111] direction, as shown in the HRTEM micrograph in Fig. 4b. Namely, the front of the surface step in the Si-whisker which is created by the leading NiSi_2_ module should be exactly perpendicular to the [111] crystallization direction, denoted by the dotted line, however in atomic scale it is jagged along the [111] and [111] directions, this is also shown schematically in the inset in Fig. 4b. These are considered as surface stapes because the material is free of strain or planar and linear defects. It is worth noting that the surface steps remain intact during the TEM specimen preparation because the specimen was not mechanically thinned, instead it was etched by HF as discussed in §1.

**Figure 5:**
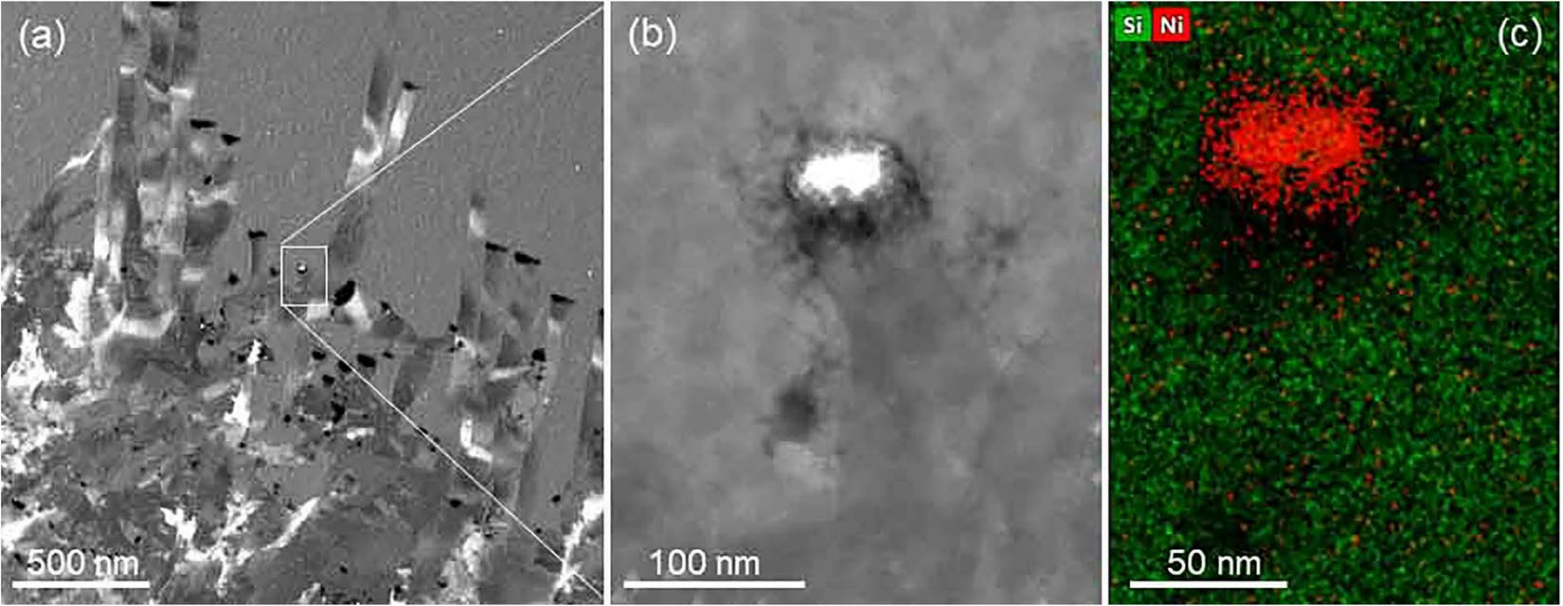
(**a**) HAADF image taken at low-mag showing many crystallized silicon whiskers. (**b**) A whisker is shown in which the leading NiSi_2_ grain is preserved and shows high contrast due to the high Z, (**c**) nickel and silicon EDS elemental maps.

The whiskers grown out from the Ni pad of this sample have been investigated by analytical methods as well. A big part of them is shown in Fig. 5a via a STEM HAADF image. Most of the tips of the whiskers are dark thanks to small holes, where the NiSi2 grain was lost from chemical etching of the TEM specimen. Although the silicide grain shown in Fig. 5b is not completely intact somehow still was preserved at its original place. The bright contrast is obtained due to the higher density and larger mean atomic number of the NiSi_2_ grain that gives intense inelastic scattering detected in HAADF mode. Ni and silicon elemental maps are recorded as well as shown in Fig. 5c proving that we still have a NiSi_2_ grain at the tip of the growing crystalline silicon whisker.

In conventional Ni-MILC where SPC is also involved, the system crystallizes the maximum number of Si atoms using the existing whiskers as seeds in order to reduce its free energy according to the minimum action principle, even forming defects, as revealed by the mosaic structure shown in Fig. 2. On the contrary, pure Ni-MILC at 413 °C is a slow process limited by Ni diffusion, followed by Si bond rearrangement at the backside of the existing NiSi_2_ lattice of the modules. The quality of this NiSi_2_ lattice is very good because it is formed at 413 °C, considering that NiSi_2_ already forms as low as 250 °C. Therefore, the process could be considered as “internal” Molecular Beam Epitaxy (MBE). Similarly the low temperature growth of a high quality epitaxial gold film on Si substrate by diffusion was demonstrated in spite of the 25% misfit between the two phases. In this experiment a 20 nm polycrystalline gold film was deposited on a (001) Si-wafer holding a 2.5 μm thick 3C-SiC epitaxial film. Upon annealing at 500 °C for 20 days a nearly perfect epitaxial gold film formed at the Si/SiC interface from the gold slowly diffusing through the SiC layer^20^.

### Poly-Si films formed by Ni-MILC at 454 °C

**Figure 6:**
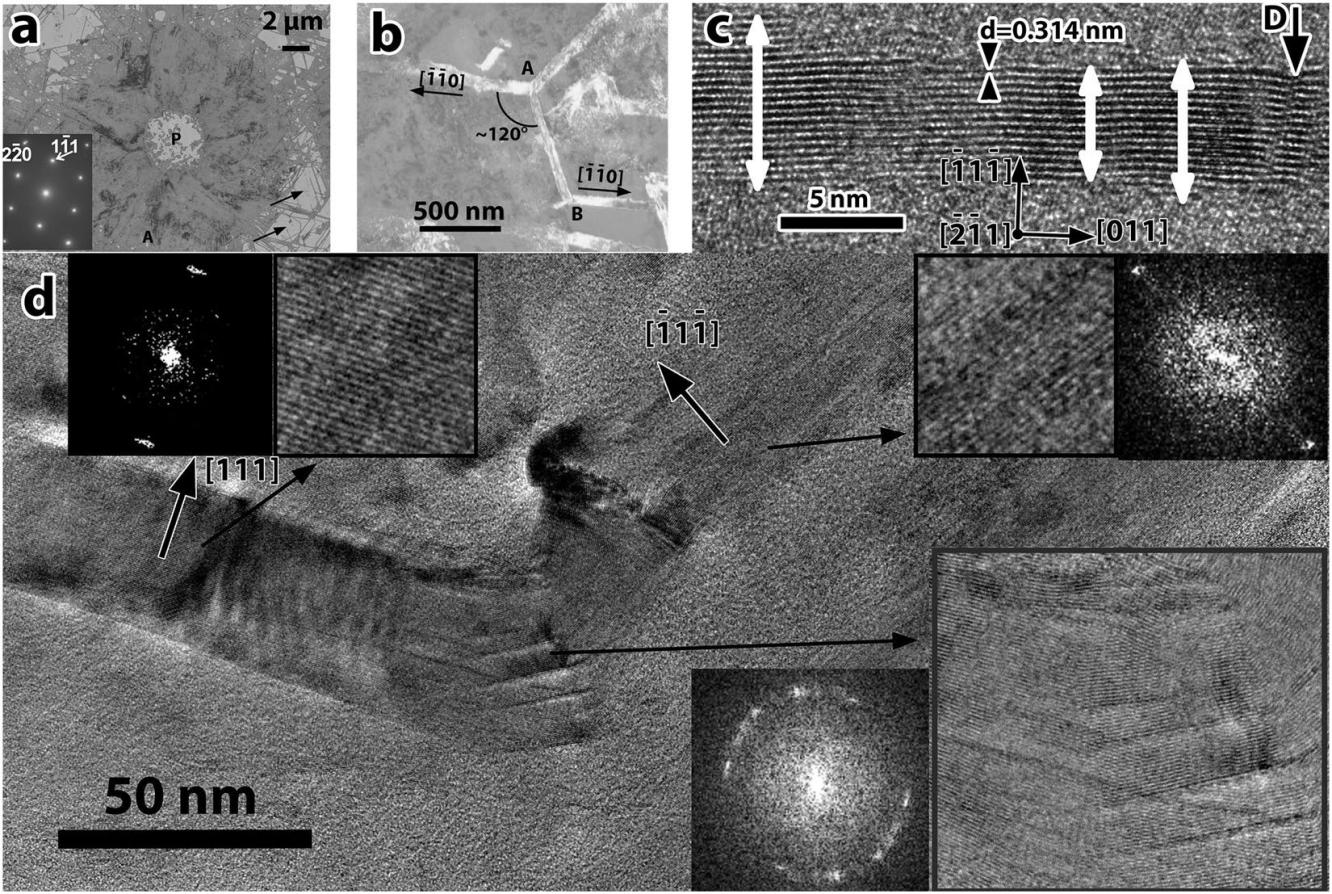
PVTEM micrographs after Ni-MILC at 454 °C for 3 Ds. (**a**) Overall view of the poly-Si around a NiSi_2_ pad. In the inset the diffraction pattern taken from the grain confirms the (110) section of this grain. Isolated whiskers are denoted by arrows at the edges of the polycrystalline area. (**b**) Three “slow” whiskers in (111) section, run along the three equivalent <110> directions. (**c**) Whisker of [011] type in (211) section, exhibiting significant width variations in spite the stable {111} sidewalls. Defects are also denoted by the letter D. (**d**) Change of the orientation of a whisker to an arbitrary direction not defined by the crystallographic characteristics of the whisker. The original and the final segments of the whisker, as well as the area of change are shown by FFTs and HRTEM micrographs.

The morphology and the mode of growth of the poly-Si films was also studied at 454 °C after 3 Ds annealing. The overall view of the poly-Si around a NiSi_2_ pad is shown in the low magnification PVTEM micrograph in Fig. 6a. The diffraction pattern in the inset was taken from the grain denoted by the letter A, revealing that it is in (110) section. In non-completely crystallized areas at the edges of the film isolated whiskers are evident denoted by arrows. The NiSi_2_ pad is denoted by the letter P, it is almost empty because the NiSi_2_ was etched by the HF during the PVTEM specimen preparation. Slow random type whiskers were also observed at this temperature, as shown in Fig. 6b revealing that the uniform poly-Si films consist of a mixture of fast and slow whiskers confirming the SPC suppression. In respect of the film crystallized at 413 °C, the increase of the temperature by 41 degrees has increased the crystallization rate more than one order of magnitude. However, the quality of the whiskers is inferior in respect of the uniformity and the defect density, suggesting a weak contribution of SPC at 454 °C. This is evident in the isolated whisker shown in Fig. 6c, which is a slow whisker of [110] type in (211) section. The sidewalls are faceted by {111} planes. These planes belong to the Si-crystal equilibrium shape and the sidebands should be regular and smooth. In contrary the width of the whisker varies from place to place, also defects inside the crystal were formed, denoted by the letter D. It is evident that even at the low temperature of 454 °C some SPC is involved in MILC process.

In some cases, the whiskers change direction non-related with the structural characteristics of the whisker. This is the case of the whisker in Fig. 6d which exhibits 111 lattice planes parallel with the whisker axis on both sides of the kink in the middle of the image. The orientation difference between the two sections is 55° which is far from the angle between equivalent {111} type planes. (70.5°). In Fig. 6d the Fast Fourier Transform (FFT) and the HRTEM are shown in the insets before and after the change of the growth direction.

### Nickel-disilicide inclusions

**Figure 7:**
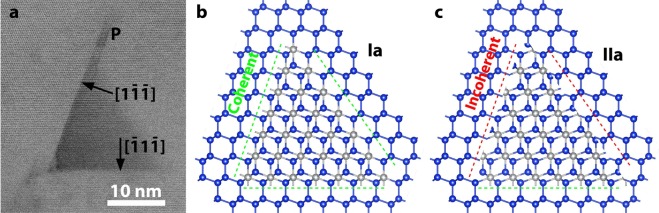
Lattice image of a coherent NiSi_2_ inclusion embedded in the Si matrix. (**a**) STEM-BF micrograph at high magnification of a NiSi_2_ tetrahedral inclusion in a whisker grown along the [111] direction in (110) section. From the upper edge of the tetrahedral inclusion a plate-like inclusion is extended bounded by {111} planes, denoted by the letter P. (**b**) Schematic representation of a tetrahedral NiSi_2_ inclusion having four Ia type interfaces with the Si matrix. (**c**) Model of an inclusion having one IIa type interface. In this case only the base is coherent, all the others sidewalls are incoherent.

Although the defect density in whiskers grown at 413 °C is very low^5^, a new V-shape defect was observed, not related to linear or planar defects, denoted by the letter T in the micrograph in Fig. 4a. The V-shape defects have a maximum size of 20 nm, and their boundaries are coinciding with the {111} lattices planes. These defects were systematically studied by Radnóczi *et al*.^21^ by high resolution STEM in High Angle Annular Dark Field (HAADF) mode, which is sensitive to Z-contrast, also by Electron Energy Loss Spectroscopy (EELS) in a specimen annealed at 413 °C for 11 Ds and 442 °C for additional 11 Ds. It was shown that they are coherent NiSi_2_ tetrahedral inclusions, which due to the 0.4% misfit with Si and their small size cannot create misfit dislocations^21^. The tetrahedral NiSi_2_ inclusion appears as triangle viewed in (110) section as shown in the high magnification STEM-BF micrograph in Fig. 7a. A plate-like inclusion emanates also from the tetrahedral NiSi_2_ bounded by {111} planes, denoted by the letter P in Fig. 7a. The inclusion is coherent with the Si matrix. For stress relief by misfit dislocations a dislocation of the 1/2[110] type should be formed in every 92 nm; while the size of the inclusions is of the order of 20 nm only.

The coherent (111) type NiSi_2_/Si interfaces were systematically studied by Cherns *et al*.^22^. They can appear in four variants Ia, Ib, IIa, IIb, where (a) and (b) are distinguished by the silicide being terminated on planes of silicon or nickel atoms, respectively. The model II is simply related to the model I by rotation of the silicide by 180° on the [111] axis^22^. The requirement a tetrahedral NiSi_2_ inclusion embedded into silicon matrix to be coherent is satisfied only for the variants Ia, Ib, as schematically shown in Fig. 7b. On the contrary, the variants IIa and IIb can be coherent only in one of the sidewalls of the tetrahedron, while being incoherent in all the others as shown in Fig. 7c. It was distinguished which of the two coherent variants Ia and Ib is preferable by taking High Resolution-HAADF micrographs from the boundaries of the embedded tetrahedral inclusions in the exact (110) section. Thanks to the high resolution Z-contrast imaging, the columns of Ni and Si atoms viewed edge on were distinguished revealing that the type Ia configuration is preferable, see Fig. 9 in ref.^21^. It is speculated that the variant Ia is preferable in respect of the Ib due to the lower number of dangling bonds. Namely, every Ni atom at the interface has one dangling bond in the case of variant Ia, instead of two as in the case of Ib ref.^22^, consequently the variant Ia is more stable than the Ib.

Nickel-disilicide inclusions having octahedral, tetrahedral and (111) plate-like shape were also found in bulk single crystal Si intentionally contaminated by Ni. These are formed upon cooling to room temperature even after quenching, in contrary to other impurities which remain supersaturated as interstitial, for example Fe^23,24^. The easy precipitation of NiSi_2_ in Si is attributed to the very low misfit between them. The formation of NiSi_2_ precipitates in Si is used for the trapping other metallic impurities there. This is a novel pathway for engineering impurities in Si^25^.

The Ni solubility in Si at 600 °C is very low, of the order of 10^13^ atoms/cm^3^ concentration^26^. However, the Ni concentration in poly-Si films grown by Ni-MILC at this temperature is about six orders of magnitude higher, 4 × 10^19^ atoms/cm^3^ or 0.08 atomic % of Ni, as Secondary Ion Mass Spectroscopy (SIMS) reveals^9,27^. As far as we know, the incorporation of the excess Ni in conventional Ni-MILC at 600 °C is not known. Due to the mosaic structure it is speculated that the excess Ni is incorporated at the low angle boundaries of the sub-grains. For Ni-MILC occurring below 450 °C the excess Ni is trapped in the tetrahedral inclusions in the form of the NiSi_2_. The inclusions are homogeneously distributed along the whiskers and the Ni percentage incorporated in these is about 0.04 at%^20^. The lower Ni concentration is expected due to the lower formation temperature and it was predicted by theoretical calculations on Ni-MILC modeling^9^. In the case of samples annealed at 413 °C the Ni residual concentration was also determined and a value of 0.032 at% was obtained. See details in Supplementary info of this paper. It was shown that the Ni residual concentration is increasing with increasing annealing temperature, and is not depending too much on annealing time.

#### Experimental procedure

For the Ni-MILC experiment a glass substrate (Corning Code 7059) was covered with a 200 nm SiO_2_ buffer layer grown by Plasma Enhanced Chemical Vapor Deposition (PECVD). A 50 nm thick intrinsic a-Si film was deposited on the buffer layer by Low-Pressure Chemical Vapor Deposition (LPCVD) at 500 °C using silane. Then a 50 nm thick silicon oxide capping layer was deposited by Plasma Enhanced Chemical Vapor Deposition (PECVD). For the Ni-MILC process, windows were opened by lithography and etching on the upper oxide protection layer. Subsequently a 15 nm thick nickel film was deposited on top by vacuum evaporation in RT. The thickness of the Ni film was chosen to give stoichiometric NiSi_2_ to all the depth of the pad after annealing at 250 °C for 10 minutes in nitrogen atmosphere and NiSi_2_ pads were formed. After the formation of NiSi_2_ at the pads, the remnant of the nickel layer was washed off with nitric acid. In this way periodic NiSi_2_ pads with different size were formed. Subsequently rectangular pieces with dimensions of 5 mm × 15 mm were cut from this wafer and placed in quartz ampoules, which were sealed in vacuum at 8 × 10^−2^ (Pa) and placed in furnaces for long annealing at 413 °C for 11 Ds (days), also for 32 Ds, respectively. Conventional MILC specimens were annealed at 600 °C for 1 hour in nitrogen atmosphere for reference purposes. Specimens for Plane View TEM (PVTEM) observations were prepared by etching the upper-level protection SiO_2_ layer, the SiO_2_ buffer layer and the glass substrate using HF and subsequently lifting off the Si film on gold micro-grids.

For the structural characterization a 2010 JEM microscope was used. Also for High Resolution Scanning-TEM (HRSTEM) imaging a FEI Titan G2 60–300 microscope was used having ~70 pm point resolution, equipped with Gatan GIF Quantum Dual Electron Energy Loss Spectrometer (EELS). For EDS measurements an FEI THEMIS 200 TEM/STEM was used.

## Conclusions

The structural characteristics of the poly-Si films grown by Ni-MILC at temperatures >500 °C are compared to those grown at 413 °C and are summarized in the Table 1.

**Table 1:** Characteristics of the poly-Si films crystallized by Ni-MILC.

	Conventional Ni-MILC	Low Temperature Ni-MILC
Range of Temperature	600 °C > T > 500 °C	T = 413 °C
Ni-MILC process	SPC is involved	No SPC is involved
Morphology of the film	Mosaic	Tweed-like
In-grain defects	High density of Low-Angle Boundaries.	Only a few Stacking Faults NiSi_2_ inclusions
Grain boundaries	Very irregular.	Smooth or saw-tooth in <111> type whiskers
Whiskers	Only [111] type (fast)	Fast [111] type. Slow in any other directions
Change the whisker direction	Frequent	Rare
Ni atomic % concentration	0.08^9,27^	0.032
NiSi_2_ inclusion	Invisible	Visible, mainly tetrahedral
Annealing time	Short	Very long

The contribution of the SPC during the Ni-MILC process for temperatures above 500 °C was clearly shown by TEM observations. The SPC contribution is significant because the long incubation period which is required for SPC to start is abolished due to the formation of fast growing <111> whiskers acting as nucleation centers. The SPC is completely suppressed and pure Ni-MILC occurred at 413 °C. Fast and slow growing whiskers mainly contribute to the formation of the uniform poly-Si film, which for this reason has a tweed-like structure. The <111> whiskers exhibit a saw-tooth faceting in their sidewalls similar to those observed in nano-wires grown by the VLS method. The whiskers have a low defect density, therefore the uniform poly-Si which is produced by pure MILC at 413 °C is superior to that produced by the standard MILC above 500 °C where SPC is involved resulting in mosaic structure.
